# Pollen genotyping by SABER–MassARRAY reveals that fewer than half of honey bee visits can cross-pollinate a self-incompatible crop

**DOI:** 10.1098/rspb.2025.0891

**Published:** 2025-06-25

**Authors:** Wiebke Kämper, Helen M. Wallace, Stephen J. Trueman

**Affiliations:** ^1^ Functional Agrobiodiversity & Agroecology, University of Göttingen, Göttingen, Germany; ^2^ Queensland University of Technology, Brisbane, Queensland, Australia; ^3^ Griffith University—Nathan Campus, Brisbane, Queensland, Australia

**Keywords:** *Macadamia integrifolia*, NGS, pollen flow, cross-pollination, pollen limitation, Proteaceae

## Abstract

Pollen limitation occurs when insufficient pollen or the wrong pollen genotype reaches the stigma. The pollination efficiency of flower visitors to crops has been investigated, but the genotypes of crop pollen carried have rarely been identified. We developed a method that detects SNPs in the pollen carried by single bees, using a customized single allele base extension reaction (SABER) with MassARRAY to distinguish genotypes that contribute only a small fraction to a mixed-genotype pollen sample. We used this method to identify the cultivars of pollen carried by honeybees at increasing distances from a cross-pollen source in two multi-cultivar macadamia orchards, one with wide single-cultivar blocks and one with narrow single-cultivar blocks. We found that many honeybees carried exclusively self-pollen. Only 30–53% of honeybees carried cross-pollen, representing the maximum that potentially contributes to crop production in self-incompatible crops. Distance from a cross-pollen source or the orchard design did not significantly affect the percentage of honeybees carrying cross-pollen. This study demonstrates significant potential to increase the effectiveness of honeybees as pollinators. Orchards can be re-designed to interplant cross-pollen sources and maximize the number of honeybees contributing to crop production. Improving pollination effectiveness will help to alleviate the growing shortfall in the supply of beehives required for crop pollination.

## 1. Background

Many temperate and tropical crops experience pollination deficits that lead to suboptimal food production [1]. The level of pollen limitation is defined as the difference between the maximum yield achievable following optimal pollination and the observed yield [2]. More than 60% of all plants are thought to be pollen limited and reproduction increases by 63%, on average, following pollen supplementation by hand [3,4]. Pollen limitation has two possible components, pollen quantity and pollen quality, that can affect plant reproductive output [5]. The quantity of pollen deposited on the stigma is not always the cause of pollen limitation, as the quality of deposited pollen may also be important [5–8]. An effective pollinator must transport conspecific pollen from the anthers to the stigmas to effect pollination, but more importantly, the pollen genotypes deposited on the stigma must be capable of causing fertilization and seed development [9,10]. Conspecific pollen deposition does not always lead to fertilization, as genetic incompatibility mechanisms in the flower can prevent pollen germination on the stigma, pollen tube growth through the style, or pollen tube penetration of the ovules [11–13]. Late-acting incompatibility in the embryo sac or the developing embryo, or the selective abortion of inbred fruitlets, can also prevent some genotypes of conspecific pollen from producing mature, harvestable fruit [14].

The production of seeds in self-incompatible crops depends on cross-pollination [8,15–18]. Furthermore, the production and quality of fruit or seeds in many self-compatible crops benefit from cross-pollination [5,17,19,20]. In clonally propagated tree and shrub crops, all the plants of one cultivar are genetically identical (i.e. they are the same genotype) and so cross-pollination requires that pollen from a different cultivar is transferred to the stigma. However, the closest trees of a different cultivar might be located far from the flowers, because farms are often established with wide blocks of single cultivars to optimize the efficiency of pest and disease management, irrigation scheduling, fertilizer application and harvesting [21–24]. In animal-pollinated crops, flower visitors often need to travel long distances across single-cultivar blocks to visit trees of more than one cultivar during a single foraging trip and, if they fail to do so, they may be unable to cross-pollinate a self-incompatible crop [25].

Pollinators do not appear to transport large amounts of cross-pollen over long distances across orchards of tree crops such as apple, avocado, chestnut, macadamia and mango [7,26–31]. honeybees, which are the main pollinators of many crops globally, are often unlikely to travel across multiple rows of an orchard to collect floral resources [10,32], especially in mass-flowering crops like macadamia where each tree can produce 100 000−400 000 flowers in just a few weeks each year [30,33,34]. About half of the honeybees forage in the row right next to their hive in macadamia orchards [32], and honeybees often forage along rows rather than across rows in apple and almond orchards [26,35]. This type of along-row foraging minimizes the potential for cross-pollen transfer if the orchards contain only one cultivar within each row. Planting pollinizer trees is sometimes unpopular among orchardists because it can reduce the total yield, average quality or marketability of the harvested fruit if the pollinizer trees have lower yield, quality or marketability than the main cultivar. Moreover, planting pollinizer trees is often considered unnecessary when the orchard is established with two main cultivars in alternating rows [23,36], although this assumption may be false if honeybees rarely forage across the rows [37,38]. Understanding the movement patterns and the genotypes of pollen carried by pollinators is important to maximize compatible-pollen transfer and to optimize the proportion of pollinator visits that can lead to fruit set and crop production. This is especially important now that wild bees are declining in abundance and diversity, and there are shortfalls in the supply of honeybee hives needed for crop pollination [15,39–42].

Identifying the species or cultivars of pollen carried by pollinators has traditionally required tedious and highly skilled microscopic examination of pollen grain morphology [43–46], if cultivar identification was possible at all. Identifying the species of pollen has become more rapid and convenient with the development of DNA metabarcoding, which allows matching of DNA-sequence reads from the pollen against large DNA-sequence libraries that have been compiled from species across the plant kingdom [45,47–49]. However, identifying the cultivars of crop pollen using DNA sequencing is technically more challenging, as it requires both (i) a DNA-sequence library for the different cultivars within a species and (ii) a method to distinguish DNA sequences of low-abundance cultivars among the high-abundance cultivar(s) in a mixed-cultivar pollen sample. To our knowledge, we are the first to analyse pollen cultivars using advanced molecular tools to discern the percentage of honeybees that can effectively pollinate flowers among the total population of foraging honeybees.

In this study, we aimed to identify the cultivars of pollen carried by honey bees at different distances from a cross-pollen source. To do this, we customized a single allele base extension reaction (SABER) technique that can discriminate low-abundance DNA sequences with rare or mutant single-nucleotide polymorphisms (SNPs) among a background of high-abundance DNA sequences with a common or wildtype SNP [50,51]. SABER was developed originally to identify rare SNPs in human foetal DNA among the predominant maternal DNA in a human maternal plasma sample, without the need for more invasive procedures such as amniocentesis or chorionic villus sampling [50]. Here, the technique allowed us to identify each cultivar in a pollen sample obtained from a single honey bee foraging on macadamia (*Macadamia integrifolia* Maiden & Betche) flowers. Macadamia flowers have a pollen presentation mechanism in which the upper style dislodges pollen from the anthers during anthesis, the anthers are then curled back underneath the four perianth segments, and self-pollen is presented on the upper style and stigma [52,53]. This may appear to be a mechanism that promotes self-fertilization. However, bees can remove much of the self-pollen and deposit cross-pollen before the stigma becomes receptive [52,54]. Macadamia, in fact, has a realized mating system that is highly outcrossing [55], resulting from greater pollen tube growth following cross-pollination than self-pollination [56,57] and from abscission of most of the self-pollinated fruitlets during the early stages of fruit development [58].

We aimed to determine the percentage of honey bees carrying cross-pollen (i.e. pollen genotypes different from the tree where the honey bee was collected) at increasing distances from a cross-pollen source in two macadamia orchards with different designs. This value corresponds to the maximum percentage of honey bee visits to individual flowers that potentially contribute to cross-pollination in a self-incompatible crop such as macadamia [55,58]. The results of this study can be used to design orchards and distribute beehives in ways that maximize the number and efficiency of honey bees contributing to crop production.

## 2. Material and methods

### (a) Sample sites and collection

**Figure 1.**
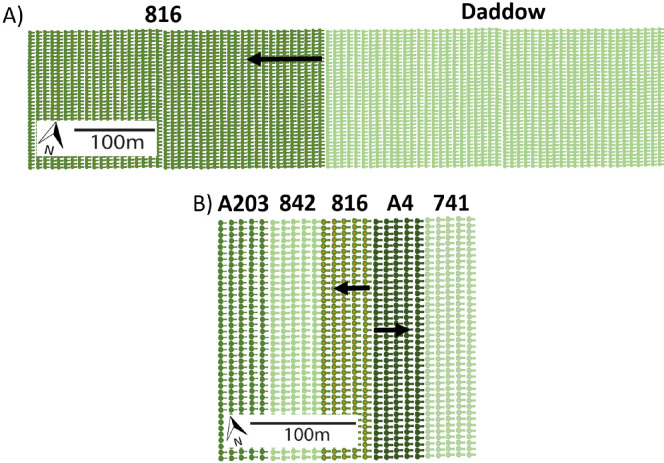
Macadamia orchard designs: (A) A ‘wide-block orchard’ consisting of 42 rows of a single cultivar (‘816’) planted next to 48 rows of another single cultivar (‘Daddow’), and (B) a ‘narrow-block orchard’ consisting of single-cultivar blocks with each cultivar planted in five contiguous rows. Black arrows show an example of (A) one transect across a wide block of cultivar ‘816’, (B) one transect across a narrow block of cultivar ‘816’, and one transect across a narrow block of cultivar ‘A4’. A transect consisted of individual trees in: (A) the first row (8 m), second row (16 m), third row (24 m) and eleventh row (88 m) in the wide-block orchard, and (B) the first row (10 m), second row (20 m), third row (30 m) and fourth row (transects across 816: 40 m from ‘A4’; but 20 m from ‘842’; transects across A4: 40 m from ‘816’; but 20 m from ‘741’) in the narrow-block orchard.

We sampled honeybees from two commercial macadamia orchards near Bundaberg, Queensland, Australia (24°47′54″ S, 152°17′36″ E, and 24°56′7″ S, 152°21′16″ E), which were 16 km from each other. Cultivars in the first orchard (hereafter: the wide-block orchard) were planted in wide single-cultivar blocks, with 42 rows of cultivar ‘816’ planted next to 48 rows of cultivar ‘Daddow’ (figure 1A). A few storm-damaged ‘816’ trees in this orchard had been replaced with trees of cultivar ‘741’. The nearest other macadamia trees were additional rows of ‘816’ and ‘Daddow’ that were planted immediately WSW of the wide ‘816’ block. The trees were 10 years old, and tree spacing was 8 m between rows and 4 m within rows. The orchard covers ~80 ha. Western honeybee (*Apis mellifera* L.) hives were placed around the orchard during flowering at a stocking rate of 5 hives per ha, consistent with the recommended rate for Australian macadamia orchards of 5−8 hives per ha [59].

Cultivars in the second orchard (hereafter: the narrow-block orchard) were planted in narrow single-cultivar blocks, each five rows wide, with blocks of cultivars ‘A203’, ‘842’, ‘816’, ‘A4’ and ‘741’ planted next to each other (figure 1B). The nearest other cultivars were ‘A16’, ‘A38’, ‘A268’, ‘Daddow’, ‘Own Venture’, ‘814’, ‘842’ and ‘849’, located at least 300 m away. The trees were 13 years old, and tree spacing was 10 m between rows and 2 m within rows. The orchard covers ~250 ha. Western honey bee hives were also placed around this orchard during flowering at a stocking rate of 5 hives per ha.

Honey bees were collected along transects within each orchard. Each transect proceeded perpendicularly into a single-cultivar block from the edge row of another cultivar. A transect in the wide-block orchard consisted of individual trees in the first row (8 m), second row (16 m), third row (24 m) and eleventh row (88 m) from the cross-pollen source. A transect in the narrow-block orchard consisted of individual trees in the first row (10 m), second row (20 m), third row (30 m) and fourth row (40 m; but 20 m from the single-cultivar block on the other side) from one of the cross-pollen sources. Honey bees were collected from eight transects in the wide-block orchard, with all eight transects located in cultivar ‘816’ (figure 1A), and from seven transects in the narrow-block orchard, with four transects located in cultivar ‘816’ and three transects located in cultivar ‘A4’ (figure 1B). Honey bees were collected once per transect in sunny weather and almost windless conditions during peak flowering between 9 September and 12 September 2017. At each sampling point, five to six honey bees were caught with a fine mesh net during a flower visit (i.e. when they were observed to contact a macadamia flower) and immediately transferred into individual tubes filled with 80% ethanol.

### (b) Pollen genotyping

Tubes containing a honey bee and ethanol were vortexed to dislodge pollen from the body, including the corbiculae, before centrifuging the sample for 30 min at 4000 rpm. The honey bee was removed from the tube, and 100 µL of the pollen-ethanol suspension was transferred from the bottom of each tube into a fresh round-bottom tube. Tubes were placed in an incubator at 72°C until all ethanol had evaporated, before adding zirconia/silica beads and shaking on a TissueLyser II (Qiagen, Hilden, Germany). DNA was extracted using the DNeasy Plant Mini Kit (Qiagen, Hilden, Germany).

We used a MassARRAY approach that was based on unique, homozygous SNPs that we identified previously from each macadamia cultivar (table 1) [30,58]. We combined this approach with the highly sensitive SABER technique on the Agena MassARRAY platform (Agena Bioscience, San Diego, CA) to detect less-common genotypes that might contribute only a small fraction of the genotypes within a mixed pollen sample. The extracted pollen DNA was amplified using multiplex PCR reactions following our previous protocol [30] and grouping the markers according to the targeted extension base for SABER [58]. Standard PCR protocols extend both the putatively predominant allele and the alternative allele together. The SABER protocol extends only the alternative allele [50,51] which, in this case, may be provided by a less common pollen source, and then, in a separate assay, extends only the putatively predominant allele. Unincorporated dNTPs were deactivated using 0.5 U of shrimp alkaline phosphatase and keeping the sample at 37°C for 40 min and 85°C for 5 min. Primer extension was initiated by adding 1 U of iPLEX Pro, 3 pmol of the relevant acyclonucleotide (New England BioLabs, Ipswich, MA) and extension primers, and performing a reaction with initial denaturation at 94°C for 2 min, followed by 35 cycles of 94°C for 10 s, 55°C for 10 s and 70°C for 1 min, followed by final elongation at 70°C for 5 min. Cation exchange, SpectroCHIP loading and mass spectrometry were performed [30]. TYPER 4.0 software (Agena) was used to interpret mass spectra and to identify alleles for genotyping of the mixed pollen samples.

**Table 1.** Single-nucleotide polymorphisms (SNPs) identified from 12 macadamia cultivars. A ddRADseq approach was used to screen leaf samples from the 12 cultivars for private alleles (i.e. homozygous SNPs that are unique for one cultivar). Locus provides a cross-reference to the sequence surrounding the SNP (see electronic supplementary material for sequence and primer information). REF = polymorphism in most of the 12 cultivars. ALT = polymorphism in the target cultivar. The nucleotides in bold type represent the unique homozygous SNPs for each cultivar (i.e. ALT).

locus	REF	ALT	741	814	816	842	849	A4	A16	A29	A38	A203	Daddow	Own
														Venture
27 718	G	T	**TT**	GG	GG	GG	GG	GG	GG	GT	GT	GG	GG	GG
52 998	C	A	**AA**	CC	CC	CC	CC	CC	CC	CT	CT	CC	CC	CC
3756	C	T	CC	**TT**	CC	CC	CC	CC	CC	CC	CC	CC	CC	CC
10 725	T	C	TT	TC	**CC**	TT	TT	TT	TT	TT	TT	TT	TT	TT
10 814	A	G	AA	AA	AA	**GG**	AA	AA	AA	AA	AA	AA	AA	AA
13 501	G	T	GG	GG	GG	NA	**TT**	GG	GG	GG	GG	GG	GG	GG
74 523	G	T	GG	GG	NA	GG	**TT**	GG	GG	GG	GG	GG	GG	GG
24 338	G	A	GG	GG	GG	GG	GG	**AA**	GA	GA	GA	GG	GG	GG
8730	G	T	GG	GG	GG	GG	GG	**TT**	TT	GT	GT	GG	GG	GT
71 837	C	G	CC	CC	CC	CC	CC	CC	**GG**	CC	CC	CC	CC	CC
3801	C	T	CC	CC	CC	CC	CC	CC	CC	**TT**	CC	CC	CC	CC
78 400	G	A	GG	GG	GG	GG	GG	GG	GG	GG	**AA**	GG	GG	GG
88 700	C	G	CC	CC	CC	CC	CC	CC	CC	CC	**GG**	CC	CC	CC
1724	T	C	TT	TT	TT	TT	TC	TT	TT	TC	TC	**CC**	TT	TT
14 937	A	G	AA	AA	AA	AA	AA	AA	AA	AA	AA	AA	**GG**	AA
47 410	T	C	TT	TT	TT	TT	TT	TT	TT	TT	TT	TT	TT	**CC**
60 567	A	T	AA	AA	AA	AA	AA	AA	AA	AA	AA	AA	AA	**TT**
86 621	C	T	CC	CC	CC	CC	CC	CC	CC	CC	CC	CC	CC	**TT**

### (c) Data analysis

We assessed the effect of distance to another cultivar on the percentage of honey bees carrying cross-pollen. For this, we categorized those honey bees that carried any cross-pollen, namely, those that either carried only cross-pollen or both cross and self-pollen, and defined these specimens as being capable of cross-pollinating the self-incompatible crop, macadamia (score = 1). The remainder of honey bees, which exclusively carried self-pollen, were defined as not capable of successfully pollinating the crop (score = 0). We then used the scores in a GLMM with binomial distribution to infer whether the likelihood of a honey bee being able to (cross-)pollinate the flower depended on the following fixed variables: distance from another cultivar (number of rows), orchard type (wide vs narrow-orchard block), and the interaction term between distance and orchard type. The transect along which a honey bee was sampled was included as the random effect. We used R version 4.4.0 to run the R packages glmmTMB for fitting the GLMM, DHARMa for simulation-based residual diagnostics, lsmeans for pairwise comparisons, and performance to calculate *R*^2^ values [60–63]. Means were regarded as significantly different at *p* < 0.05. Data visualization was performed using SigmaPlot version 14.0 (Systat Software Inc., San Jose, CA, USA).

## 3. Results

**Figure 2.**
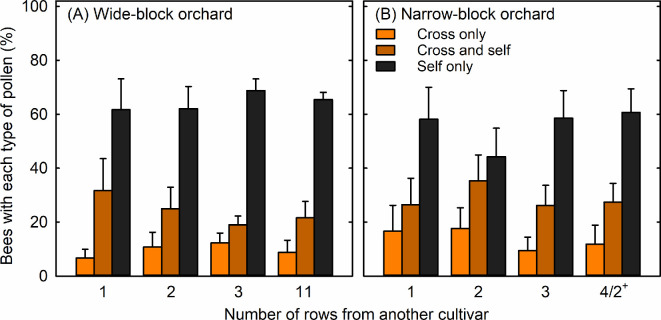
Percentage of honeybees carrying exclusively cross-pollen, both cross-pollen and self-pollen, and exclusively self-pollen at different distances from a cross-pollen source in (A) a wide-block macadamia orchard and (B) a narrow-block macadamia orchard. honeybees were collected along transects starting at trees adjacent to another cultivar (row 1) and ending at (A) 11 rows from another cultivar (row 11) for the wide-block orchard where two cultivars were planted in blocks of 42 or 48 rows, and ending at (B) the fourth row or second row in either direction from another cultivar (row 4/2^+^) for the narrow-block orchard where each cultivar was planted in five contiguous rows. Means (+ s.e.) across rows within an orchard for the percentages of honeybees carrying cross-pollen are not significantly different (GLMM, estimate = −0.17, s.e. = 0.15, *p* = 0.24, *n* = 7–8 transects).

A total of 188 and 156 honey bees were collected in the wide-block orchard and the narrow-block orchard, respectively. Only 7–12% and 10–18% of honey bees carried exclusively cross-pollen (i.e. pollen of a different cultivar than the tree cultivar from which we collected the honey bee) in the wide-block orchard and the narrow-block orchard, respectively (figure 2). Another 19–32% and 26–35% of honey bees carried both cross-pollen and self-pollen in the wide-block orchard and the narrow-block orchard, respectively (figure 2). Most honey bees, specifically 62–69% and 44–61%, carried exclusively self-pollen in the wide-block orchard and the narrow-block orchard, respectively (figure 2).

We analysed whether the likelihood of a honeybee being able to pollinate the flowers depended on the distance from another cultivar and the orchard type, after categorizing potentially effective pollinators as those honeybees that either carried only cross-pollen or both cross and self-pollen. We found that only 30–38% and 36–53% of honeybees carried cross-pollen. The distance from a cross-pollen source did not significantly affect the percentage of honeybees that carried cross-pollen (figure 2; GLMM: estimate = −0.17, s.e. = 0.15, *p* = 0.24; see electronic supplementary material, table S2 for full GLMM output). Furthermore, orchard type did not significantly affect the percentage of honeybees that carried cross-pollen (GLMM: estimate = −0.74, s.e. = 0.39, *p* = 0.06).

Most (94–95%) of those honeybees that carried cross-pollen carried only one cross-pollen cultivar. The most common cross-pollen cultivars in the wide-block orchard were ‘741’, ‘849’ and ‘Daddow’, carried by 51%, 24% and 24% of the honeybees, respectively, that carried cross-pollen. Cultivar ‘849’ was not known to be planted in this orchard, although some unidentified ‘849’ trees might have been present in the orchard, and ‘849’ trees were planted in another macadamia orchard ~1.2 km away. The most common cross-pollen cultivars identified on the honeybees that were collected from cultivar ‘816’ in the narrow-block orchard were ‘741’ and ‘A4’, carried by 57% and 30%, respectively, of the honeybees that carried cross-pollen. The most common cross-pollen cultivars identified on the honeybees that were collected from cultivar ‘A4’ in the narrow-block orchard were ‘741’, ‘816’ and ‘842’, carried by 23%, 10% and 10%, respectively, of the honeybees that carried cross-pollen. These cross-pollen cultivars were all planted in neighbouring narrow single-cultivar blocks.

## 4. Discussion

Our results revealed that only about one-third to one-half of honeybee foragers on flowers were carrying cross-pollen during the peak flowering of macadamia, which is a self-incompatible crop [55]. This means that no more than one-third to one-half of flower visits by honeybees could have led to deposition of cross-pollen on the stigma, and so less than a half of honeybee visits could have resulted in fruit production. Plant reproduction is limited by a lack of pollen in over 60% of the 1200^+^ species of flowering plants studied previously [4]. The current results suggest that pollen limitation in many agricultural production systems could be addressed by improving the efficiency of the available pollinators by, for example, increasing the number of honeybees that carry cross-pollen.

Between 62% and 69% of honeybees in the wide-block orchard and 44–61% of honeybees in the narrow-block orchard only carried pollen of the cultivar on which they were foraging. This could be because many honeybees visited only one cultivar during a single foraging trip, meaning that they may have foraged mostly along the orchard rows rather than across the orchard rows. It could also be the result of the honeybees no longer carrying cross-pollen by the time we observed them at a flower, suggesting that honeybees do not change rows frequently during a single foraging trip and that there is little cross-pollen carryover once the bees have foraged at multiple flowers within a single cultivar. Pollen carryover describes the amount of pollen that a pollinator is still carrying from the previous cultivar that it visited, even after visiting multiple flowers of the new cultivar [10,64]. The main cultivar of cross-pollen was ‘741’, identified on 51% of the honeybees that carried cross-pollen in the wide block of ‘816’, rather than pollen of the much more abundant ‘Daddow’ trees in the wide neighbouring block. Individual trees of cultivar ‘741’ were sometimes planted within the ‘816’ block to replace storm-damaged trees. Pollen was also detected from cultivar ‘849’, which was not known to be planted in the orchard, but which might have been present as individual, unidentified ‘off-type’ trees. Replacement trees, off-type trees, and rootstock shoots have recently been shown to be major contributors to cross-fertilization in many macadamia orchards [30,55]. The presence of individual trees of other genotypes might help to explain how as many as 7–12% and 10–18% of honeybees were carrying exclusively cross-pollen in the wide-block and narrow-block orchards, respectively. Most of the honeybees that carried exclusively cross-pollen were carrying pollen from cultivars other than the one in the adjoining block (i.e. they were carrying pollen from replacement trees or off-type trees that were often planted within the same row). These bees may have visited a tree of another genotype before foraging at a tree of the main cultivar and they may not yet have made contact with the pollen presenter of a flower of the main cultivar, especially if they were foraging for nectar rather than pollen [65]. Individual, and sometimes covert, cross-pollinizer trees are often located in the same orchard row as the main cultivar and they frequently underpin the levels of cross-pollination and crop production [30,55]. About half of the honeybee foragers in a macadamia orchard forage in the row immediately next to their hive [32]. A foraging pattern like this, where individual honeybees forage mostly along a single row of trees, might explain why the distance from the major cross-pollen source or orchard type did not significantly affect the proportion of honeybees carrying cross-pollen in either orchard. Many of the honeybees that were carrying cross-pollen appear to have attained this pollen from individual trees within the same orchard block rather than from trees that were across many rows in the adjoining orchard block. Poor cross-pollen transport across the rows has been observed previously in broad-acre cropping systems such as oilseed rape and in tree crop orchards such as apple, avocado, chestnut, macadamia and mango [7,26–31].

honeybees are the main pollinators of cultivated macadamia trees [53,65–68], but their movement patterns and associated pollen flow are rarely considered when introducing hives into the orchard or when planning the layout of a new orchard. We found no significant differences in the percentage of honeybees carrying cross-pollen at one row compared with 11 rows from another cultivar, and many bees carrying only self-pollen in all of the rows, indicating that the planting of single-cultivar rows may not be efficient for cross-pollination. The planting of single-cultivar rows is reducing pollination success, and interplanting multiple cultivars or individual pollinizer trees within the rows is often likely to benefit tree crops that rely heavily on honeybees for pollination [24,69]. The dependence on cross-pollen flow for almond production is already well recognized, with honeybee hives often distributed widely throughout the orchard, instead of at a few convenient locations, to ensure effective pollination [70]. In contrast, the clumping of honeybee hives rather than dispersing hives works efficiently and cost-effectively for blueberry, although only self-fertile cultivars were included in that study [71]. Almond cultivars are often still planted in an alternate-row design where each row consists of a single cultivar and the different cultivars alternate from one row to the next [8,72]. This planting design might not optimize cross-pollen deposition despite the presence of a cross-pollen source in the adjacent row. Apple and pear orchards are other examples of planting designs with single-cultivar rows, although it is common to establish a crab apple tree representing a second genotype as every tenth tree in apple orchard rows because crab apple pollen can effectively cross-pollinate apple flowers and ensure high fruit set [73].

Stable food production relies on biodiversity, including insect diversity, as fruit quality increases in the presence of wild pollinators [67,74], even if the number of managed honeybees is ample. Wild pollinator visits can result in more, larger or higher-quality products in crops such as apple, almond, cherry and strawberry, potentially as a result of unique movement patterns of wild pollinators leading to improved pollen flow across farms and an increased likelihood of cross-pollination [35,67,75–77]. Wild bees might be particularly important to consider in small farms, where they have been found to account for most of the blueberry flower visits, in contrast to large farms where the visits were dominated by honeybees [78]. Wild bees, such as mining bees and bumblebees, are more likely than honeybees to switch across rows in apple and sweet cherry orchards [10,76]. Understanding the movement patterns of honeybees and wild pollinators, and how they contribute to cross-pollen flow, is important for a wide range of tree and shrub crops that rely on cross-pollination and have similar planting schemes. This understanding would assist in the effective utilization of flower visitors, which is especially important now that wild bees are declining in abundance and diversity, and honeybee hive stocks are not keeping pace with the demand for agricultural pollination [15,40,42].

Fertilization may be hindered when the stigma is covered in self-pollen, heterospecific pollen or inviable pollen that cannot successfully fertilize the ovules [79–85]. honeybees that carry only self-pollen may cover the stigmas in self-pollen and thus reduce fertilization of macadamia ovules. Macadamia cultivars are considered partially self-incompatible in the prezygotic phase, with many self-pollen tubes arrested in the upper style before they can reach the lower style and ovary [54,56]. The initial set of macadamia fruitlets is much lower following self-pollination than cross-pollination [57,86,87]. The realized mating system among mature macadamia fruit is highly outcrossing [25,30,55,58,88,89], and pollen limitation has been demonstrated recently on a whole-tree level in macadamia orchards [30]. Arrested growth of self-pollen tubes could hinder the fertilization capacity of any cross-pollen grains that subsequently land on the stigma, potentially representing a cause of pollen limitation in addition to the limited flow of cross-pollen across the orchard rows.

The mixture of pollen genotypes carried by individual honeybees could also be the result of pollen carryover between individual flowers or of in-hive pollen transfer, instead of resulting directly from flower visits of individual honeybees to trees of multiple cultivars. Limited pollen carryover means that there is a decline in the level of cross-pollen deposition as the foraging trip progresses, as cross-pollen on the pollinator is diluted by, or replaced with, more-recently acquired self-pollen [90]. In-hive transfer describes the transfer of pollen from honeybee to honeybee, or from honeybee to surface to honeybee, within the hive that results in honeybees carrying pollen of different cultivars at the commencement of a foraging trip from the cultivars that they carried at the end of their previous trip [91,92]. Therefore, pollen carryover and in-hive transfer may lead to honeybees carrying pollen loads that do not accurately reflect the genotypes that they visited during their foraging trip. The mechanisms by which cross-pollen landed on the honeybee and the duration that cross-pollen has been on the honeybee do not matter for crop fertilization. However, understanding what leads to honeybees carrying multiple cultivars of pollen is crucial for designing smart orchards and positioning honeybee hives to improve cross-pollen flow and seed production.

Our study represents a proof-of-concept for using SABER–MassARRAY to detect a low-abundance plant genotype within a sample that may contain other plant genotypes in much higher abundance. A major advantage of the technique is that it was designed very specifically for high-sensitivity detection of very-low-abundance SNPs within a sample of much-higher-abundance SNPs [50]. The technique can be used, for example, in oncology and prenatal genetic diagnosis to detect extremely low-abundance DNA sequences from diseased or mutant cells among a much larger sample of healthy or wild-type cells [50,51,93–95]. The PCR used in the SABER only amplifies the sequence containing the low-abundance SNP, and so the MassARRAY is not affected by the abundance of other sequences in the original sample. In the current study, we used the technique to identify the cultivars of pollen on the body of a honeybee and to determine the percentage of individual visits to a flower that could be contributing to cross-pollination. Recently, we used SABER–MassARRAY to identify the paternity of young developing embryos within dissected fruitlet samples that may also contain endosperm and contaminating maternal tissue [58]. This allowed us to determine the levels of self-fertilization and cross-fertilization among very young fruitlets and to demonstrate post-zygotic genetic selection by showing that self-fertilized fruitlets were selectively aborted during later stages of fruit development. We envisage that the technique could also be used to identify the genotypes of pollen grains on a stigma or of pollen tubes at different levels down the style. This would allow fine-scale assessment of the levels of self-pollen and cross-pollen deposition on stigmas and the degree of pre-zygotic genetic selection during pollen germination and pollen tube growth.

## 5. Conclusions

SABER–MassARRAY analyses of the pollen genotypes carried by honeybees demonstrated the potential to improve the efficiency of orchard pollinators, revealing that only one in every two to three flower visits in single-cultivar blocks can possibly result in cross-pollination. The planting of large single-cultivar blocks in orchards can allow pest, disease, irrigation, fertilizer, harvesting and post-harvest processing to be managed for individual cultivars [22,23,96,97], but it can also reduce the opportunities for effective cross-pollination and high yields. For example, better cross-pollination in macadamia has the potential to increase kernel yields by around 0.5 tons per hectare and increase farm-gate income by around $US5000 per hectare [30]. Better pollination could be achieved by interplanting cultivars more closely, including by planting trees of a different cultivar within the same row as the main cultivar, or by managing honeybee pollen loads through, for example, methods to dust honeybees with cross-pollen as they leave the hive. Understanding the movement patterns of pollinators and pollen, and using this knowledge to design smart orchards, could reduce pollen limitation and increase crop production. Utilizing flower visitors effectively is especially important now that wild bees are declining in abundance and diversity, and honeybee hive stocks are not keeping pace with the demand for agricultural pollination.

## Data Availability

The data, the R script and the readme file are now available through a permanent link [98]. Supplementary material is available online [99].
